# The Power of Trading: Exploring the Value of a Trading Shop as a Health-Promoting Community Engagement Approach

**DOI:** 10.3390/ijerph17134678

**Published:** 2020-06-29

**Authors:** Lotte Prevo, Stef Kremers, Maria Jansen

**Affiliations:** 1 NUTRIM, Department of Health Promotion, Maastricht University, 6229 HA Maastricht, The Netherlands; s.kremers@maastrichtuniversity.nl; 2 CAPHRI, Department of Health Services Research, Maastricht University, 6229 HA Maastricht, The Netherlands; maria.jansen@ggdzl.nl; 3 CAPHRI, Academic Collaborative Center for Public Health, Public Health Service Southern Limburg, 6411 TE Heerlen, The Netherlands

**Keywords:** community engagement, positive health, low-SES families, partnership

## Abstract

Involving and engaging vulnerable communities from the very beginning is important if we wish to enhance general well-being. With a focus on equal partnership with low-socioeconomic status (SES) families, a Trading Shop in Vaals was developed as a community engagement initiative. In the current study, we focused on the participation process, from preparation to sustaining the Trading Shop, in order to define whether the Trading Shop can be successful in engaging families through focusing specially on their needs and perceived positive health. A formative case study design was carried out to monitor, evaluate, and timely adjust the developments within the Trading Shop by using participatory action research. The Trading Shop was monitored from its preparation to its opening, as well as during the start and the steps taken towards continuation in the form of municipal policy. The results showed one central theme during all phases: the optimal navigation between top-down support from professionals and bottom-up developments among the volunteers in the Trading Shop. With the input from both approaches, it was possible to create an optimal environment for the volunteers to achieve personal development. The inclusivity and accessibility of the Trading Shop as a community engagement initiative offered the opportunity to volunteers to enhance their needs, realizing personal growth and development of their talents in several positive health domains.

## 1. Introduction

“Alone we can do so little; together we can do so much” (Helen Keller). This famous quote grasps the essence of community engagement. Community engagement is defined as the process of working collaboratively with and through specified groups of people (communities) affiliated by geographic proximity, special interest, or similar situations to address issues affecting their well-being [1]. Creating an equal partnership with the community in health-promotion activities requires communities to get involved from the very beginning, with community members participating and taking control [2,3,4]. Particularly for vulnerable communities, such as low-socioeconomic status (SES) families, we learned that higher levels of participation are more difficult to achieve due to their living situations [5,6]. Professionals may struggle to achieve optimal communication, interaction, involvement, and exchange between the often hard-to-reach communities and professional partners [7]. This implies that, if professionals want to reach this target group, they need to firmly dive into their needs and motives to develop and implement initiatives together [3].

Community engagement practices require the finest navigation of top-down approaches, led by the expertise of professionals, as well as bottom-up approaches. The community is in charge, since they hold important local knowledge that is assumed to enhance the effectiveness and sustainability of health promotion programs [8]. The ultimate aim is the improvement of health and the reduction of health inequalities. A lengthy timeframe has to be considered, [1], where the needs and motives of the community become leading, creating a more equitable society [9]. By focusing on what really matters to the community, opportunities arise to enhance engagement among community members as a first step towards reducing the health gap [10]. Community engagement practices should also be consistent with the underlying social determinants of health, such as educational level and housing conditions [11]. In practice, this means that community engagement activities may be indirectly related to health promotion. Activities take place in several sectors, i.e., housing, social cohesion, and safety, based on the preferences of participating communities [11].

In the past years, several projects were carried out that made use of community participation activities as a key element of reaching health-promotion goals, ensuring that the needs of community members are addressed [12,13,14,15]. These projects varied in tools and methods used to achieve community engagement, such as shared decision-making, codesign, systems dynamics, and participatory interviews [12,13,14,15]. In all projects, four elements emerged that appear to be important in enhancing success. First, contextualization, in which projects must be adapted to the context local community partners operate in. Only when the community partners perceive the project as valuable, they are expected to engage more. Second, trust needs to be built during the collaboration process, creating respect and understanding among partners. Third, the accessibility of project activities for all community partners, taking, for example, language and culture into consideration. This also creates a certain feeling of fairness and equity that was found beneficial to engagement. Finally, transparency, which is especially important for professionals and governmental partners. They should communicate openly with the community partners and respond to community needs and priorities [12,13,14,15,16,17,18].

Although community engagement projects showed promising results, low-SES families remain a hard-to-engage group. This became obvious when vulnerable groups were asked to participate in projects with predefined health-promotion themes [7]. With the overall ambition of partnering with low-SES families, focusing on their own defined needs and involving them in well-being-promoting activities from the start, the Vaals Meets approach was developed. This approach in Vaals, the Netherlands, was started in July 2015. A partnership process with low-SES families aimed at their defined needs [19] led to small-scale activities [20] and, eventually, to the opening of the Trading Shop in 2018. The Trading Shop is a physical space where people can trade products and services for points instead of money. The shop also facilitates social interaction; there is a possibility to have free drinks at a communal table. The Trading Shop is run by volunteers, mostly from vulnerable groups, such as the low-SES families. The aim of the Trading Shop was to enhance community engagement. The possibility was provided to vulnerable volunteers to take part in activities in the Trading Shop based on their needs. The Trading Shop first supported volunteers to become meaningfully engaged, enhancing community participation. Secondly, the community engagement process was intended to contribute to their general well-being and positive health. The targets of the Trading Shop were based upon the concept of positive health [21], as it stands for a broader view towards health. The six domains of positive health, i.e., mental functions, bodily functions, quality of life, spiritual dimension, social participation, and daily functioning, enabled us to structure the lessons learned about the added value of the Trading Shop on the lives of these individuals [21]. Our participatory action-oriented study aimed to gain insight in the participation process of low-SES families from preparation to sustaining the Trading Shop. The value of the Trading Shop was defined by placing a special focus on satisfying the needs of low-SES families and increasing their perceived positive health.

## 2. Materials and Methods

### 2.1. Study Design

A formative case study design was carried out to monitor, evaluate, and timely adjust the developments within the Trading Shop through participatory action research (PAR) [22]. Mixed-method sources, such as quantitative data from the Trading Shop database and qualitative data from interviews with visitors and volunteers, enabled us to create feedback loops stimulating key stakeholders to optimize the community engagement process. To create an optimal environment for learning and development within the Trading Shop, a constructivist paradigm was used during the participatory research activities. An abductive process was used to abstract the key learnings of the Trading Shop to everyone involved [23].

### 2.2. Study Setting

In 2015, individuals from low-SES families were invited to take part in a photovoice study enabling them to define their needs [19]. In January 2016, the results of the needs assessment showed the desire of the families to have a physical space in the neighborhood to help fulfill their basic needs: (1) meeting each other, (2) helping each other, (3) feeling safe, and (4) being mobile. The nature of the themes showed the desire of the families to be independent and resilient, indicating that these basic needs had to be enhanced first [5,6,19]. In community engagement activities, families were supported to start organizing small-scale activities while partnering with formal and informal community partners. The feelings of independence and resilience of the families grew (see [20] for more information), probably because the support focused on more upstream determinants of health, such as people exploring their own talents or enhancing social support [24,25]. After some eighteen months of organizing, enhancing, and celebrating their small-scale community engagement activities, the families had an outspoken desire to open a Trading Shop [20].

The Trading Shop is located in the Municipality of Vaals, the Netherlands. The municipality has approximately 10,000 citizens and is defined as a moderate-to-low SES region. Although the majority of citizens have a Dutch origin, the location near the border with Germany and Belgium ensures a relatively large migration towards the Municipality of Vaals. Additionally, its citizens have an unhealthier lifestyle, more mental health issues, a poorer health status, and a shorter life expectancy compared with the average Dutch population [26,27]. During the preparation phase, from July 2017 to the opening on 17 March 2018, the Trading Shop was monitored and followed-up until the ongoing exploitation in July 2019. This study was performed in accordance with the Code of Conduct for Health Research of the Dutch Federation of Biomedical Scientific Societies.

### 2.3. Goal of the Trading Shop and Its Community Engagement Approach

Within the Trading Shop, several activities were undertaken to support the engagement of citizens and create a flourishing “trading community”. Basically, in the Trading Shop, products and services can be traded. The trading transactions work through points earned for products and services. The transactions take place without any money changing hands, with the idea of making it accessible especially for citizens in vulnerable positions. For the number of points that members of the Trading Shop had earned, they could “buy” products or services in the Trading Shop, enabling them to gain something extra that some societal groups normally cannot afford. In addition to the trading aspect, members of the Trading Shop are welcome to drink a free cup of coffee or tea at the communal table situated in the center of the shop. The community engagement activities within the Trading Shop were intended to contribute to various social objectives, such as combating the waste of products and materials, reducing poverty, and activating societal groups to participate. To this end, the targets of the Trading Shop were based on the concept of positive health [21], as it stands for a broader view towards health. The six domains of positive health, i.e., mental functions, bodily functions, quality of life, spiritual dimension, social participation, and daily functioning, enabled us to structure the community engagement processes, outputs, and outcomes [21].

With top-down support from professionals and local partners and bottom-up expertise of low-SES families and other citizens in vulnerable positions, the preparation, opening, starting, and sustainability phases of the Trading Shop were cocreated. Commitment from the families but, also, from broader community partners and citizens, was an important aspect to making the Trading Shop a success. A key factor in this process was the collaborative partnership on the level of practice, policy, and research. Under the supervision of three professionals closely involved from the beginning of the project (activation broker, participatory researcher, and policy-maker), low-SES families, together with other (vulnerable) citizens and local formal and informal partners, prepared, opened, started up, and eventually, sustained the Trading Shop for at least five more years. Over time, this collaborative partnership was put in place to monitor, evaluate, and timely adjust the development of the Trading Shop before and after the opening.

To start up the Trading Shop and make it sustainable following its starting phase, the recruitment of more low-SES families, other volunteers (mainly vulnerable groups), and formal and informal partners was needed. Solid professional support was put in place to support volunteers in managing the Trading Shop. First, this was done by the existing collaboration between the policy, practice, and research professionals. Later, with the focus on achieving policy support for sustaining the Trading Shop, a paid manager and an independent board took over (about one year after the opening). During the whole study period, the practice intermediary, a so-called activation broker [28], played a central role in encouraging vulnerable citizens to visit the Trading Shop. The broad network she had among both vulnerable groups and formal and informal partners in the community was a key factor. The Trading Shop had space for a total of 35 volunteers. During the starting phase of 15 months, a mixed group of volunteers worked in the Trading Shop (*n* = 48). Over time, a distinction became evident in a stable group of volunteers, who were retired or permanently unfit for work and wanted to stay active (*n =* 25) and a more unstable group of citizens receiving a social welfare benefit who needed activating for employment. For this so-called “exchange” group, the Trading Shop has about 10 places available, where it serves a societal goal as a next step towards employment. A close collaboration between the Municipal Social Service and the Trading Shop was in place to activate social welfare recipients.

To make the Trading Shop a sustainable initiative over time, and to assess whether municipal subsidies could be granted and become part of the local policy, the Trading Shop was carefully monitored and evaluated using feedback loops. Insights in the community engagement processes, its outputs, and outcomes were collected in interim reports for the municipality. These reports were used with the aim of making the Trading Shop part of the municipal policy with some financing.

### 2.4. Data Collection, Instrument, and Measurements

The data collected for this study had a mixed-methods nature, with the phases being monitored and evaluated: preparation (July 2015–16 March 2018) with opening (17 March 2018), starting (18 March 2018–July 2019), and continuation for municipal support in policy plans (an ongoing process). The quantitative data within this study concerned were retrieved from the monitoring system of the Trading Shop, such as number of Trading Shop members, their characteristics, and number of trading transactions of products and services of members. Next to providing an overview of members of the Trading Shop, this information was seen valuable to estimate how many low-SES families, defined as our main target group, were supported by the Trading Shop activities. Besides, these numbers gave an indication of the intensity with which the Trading Shop was used by members, gaining insight in how the Trading Shop engaged the community.

Additionally, different types of qualitative research instruments for gathering data were used. First, during all participatory activities of the Trading Shop, notes, minutes, and observations were made by the researcher and discussed with the families, policy-maker, and activation broker to instantly optimize the community participation process. From the starting phase, the Trading Shop was observed systematically by the researcher for 21 workdays (4 h) over a period of 15 months (Table 1). A focus was placed on the processes that occurred among the management/board, the level of turnover of volunteers (and reasons why), and visitors to the Trading Shop. Second, during the starting phase, semi-structured interviews with randomly selected unique visitors/members (*n* = 213) of the Trading Shop were carried out four times per year to explore the reasons for visiting the Trading Shop, how long and how often the shop was visited, what they liked about the shop, and if they had points for improvement (Table 2). The researcher was there during these observations and interviews with members/visitors present in the shop and, therefore, able to just ask members/visitors to take part in these 5-min interviews. Nobody refused to have a short conversation about the Trading Shop. Third, during the starting phase, three semi-structured interview rounds took place (twice a year) with all volunteers of the Trading Shop. An interview took about 30 min and was carried out using the positive health conversation tool, reflecting on the six domains, i.e., mental functions, bodily functions, quality of life, spiritual dimension, social participation, and daily functioning [21], in relation to the community participation in the Trading Shop. Since the conversation tool was no validated instrument, the use of it was only helpful to gain more information about the participation of the volunteers and their personal development in their overall well-being. Forty-five unique volunteers were interviewed individually, 20 of whom took part in all three interview rounds. In addition to the general questions concerning when they had started as volunteers, for how many days per week they worked in the Trading Shop, what their tasks were at that point, what they liked, and the points for improvement they saw, we asked them whether the Trading Shop actually added something to their lives (Table 3). The six domains and related questions of positive health were used as a conversation tool to actually identify their current state, helping us to, together, set a target for them to work on in the Trading Shop. Finally, twice during the starting phase, we had a two-hour meetings for all volunteers and supporting “professionals” to briefly discuss success factors and points for improvement to together decide upon what to improve in the Trading Shop in the forthcoming six months (Table 4). At the first meeting, the policy-maker, researcher, and practice worker were present, but later, their roles in the management aspect were filled by the Trading Shop Board and manager. The researcher took minutes of these meetings. The meeting was concluded by a celebration dinner.

### 2.5. Data Processing and Analysis

All quantitative data was able to be extracted from the Trading Shop database “Cyclos” and exported into Excel sheets. These Excel sheets were then imported into SPSS so that the background characteristics of all members and volunteers of the Trading Shop could be calculated with descriptive statistics and frequencies. Information about the number of trading transactions was able to be retrieved directly from the database by predesigned application programming interfaces (APIs). Next, the qualitative data gathered from notes of observations and minutes of meetings were written down in Word files. Individual interviews and group interviews were recorded and transcribed verbatim afterwards. All data, almost 700 written pages in Word files, were organized together using NVivo software to gain insight in the process, output, and outcomes of the Trading Shop for each phase. Since an abductive process was used to analyze the data, we did not start completely inductive or deductive but worked somewhat in the middle. Indicating that our previous experiences and the current state of research in the field made us sensitive for some themes in the data—for example, the positive health domains. However, these themes were not strictly defined from the beginning, enabling us to start with a rather open view towards the data [23]. First, open coding was used to select important information in the data, followed by axial coding to compare the coding and create the main themes. The overarching successes and points for improvement were always fed back to the volunteers and the management of the Trading Shop as input for the upcoming period, creating feedback loops to improve the process.

## 3. Results

### 3.1. The Preparation of the Trading Shop

**Figure 1:**
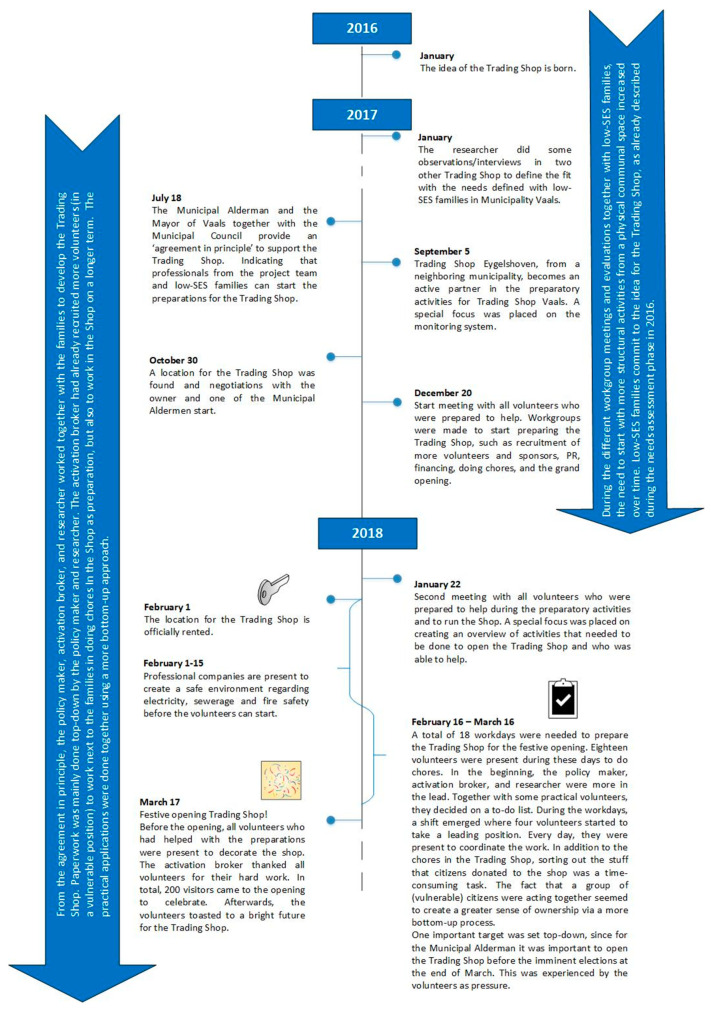
Timeline preparation process.

As a first step in the preparation for the opening of the Trading Shop, a lot of paperwork had to be done. While input for the Trading Shop was given by the low-SES families, the leading professionals (policy-maker, activation broker, and researcher) managed the paperwork. The researcher collected the “best practice” through observations and interviews with key stakeholders in two other existing Trading Shops. This was helpful in defining the fit that the Trading Shop would have with the targets of Vaals Meets and those of the participating families. It also provided some first-hand input for the adaptations that we needed to create a better fit. After the principle agreement from the municipality and the commitment of the location, the families and other, mainly vulnerable, volunteers were included from the start of the preparations for the festive opening of the shop. Sufficient numbers of community partners (*n* = 18) were committed to supporting the Trading Shop in terms of products or money, and sufficient numbers of volunteers (vulnerable citizens/families) (*n* = 30) were available to do chores in the Trading Shop in preparation for the opening. Although the deadline for the opening was set top-down, the preparatory actions developed more bottom-up, with the festive opening as a success (See Figure 1).

### 3.2. The Starting Phase of Trading Shop Vaals

#### 3.2.1. Engagement by Volunteers

A total of 63 volunteers contributed actively to the Trading Shop. Thirty volunteers assisted during the preparation of the Trading Shop—of whom, 12 continued volunteer work in the Trading Shop after the opening and were still active after the first 15 months. The Trading Shop has—and needs—about 35 active volunteers to keep it going. From preparation to the starting phase, 28 volunteers stopped volunteering due to multiple reasons, such as having a paid job or starting a procedure to get this job (*n* = 15), having different volunteer work (*n* = 4), and having physical and/or mental complaints hindering their work (*n* = 7). The main purpose for volunteering was to become or stay socially active, have something meaningful to do, and gain some work experience. For some volunteers, the provision of structure to their day/life was important, just as the possibility to learn to deal with others and have more personal contacts. Other volunteers needed to be at the Trading Shop to stay vital and active.

During the individual interviews, about two-thirds of the volunteers mentioned working one day per week, while the other one-third worked two days a week. Due to the rotation schedule, volunteers learned different tasks, such as the intake of products, checkout, clothing corner, host, and stockroom. Some volunteers were not capable of doing all tasks, which was taken into account by the management. Although all volunteers mentioned having some preferred tasks, the variation due to the rotation scheme was appreciated. All volunteers stated that the Trading Shop was a good initiative that was also beneficial to citizens of Vaals. Particularly, the possibility to trade products and services and being a meeting place for citizens of Vaals was mentioned as contributing to society. For themselves, volunteers mentioned being among “the people”, having this social contact, and the feeling that they can add something valuable to others as the added value of the Trading Shop.

“I still think it’s important to give something back and do something meaningful for society. And I also believe in sustainability and not just disposable products that can still be used. Yes and of course also the contacts. I have met quite a few people here, so in that sense it brings me some satisfaction.”

Volunteers explained that the Trading Shop mostly contributed to the positive health domain of social participation. All volunteers stated that the most valued contribution of the Trading Shop concerned enabling them to meet other people. They appreciated the social contacts they had with other volunteers and visitors. Some also said that, before they started as volunteers, they had felt relatively isolated, making the Trading Shop a first step to a more active participation in society. Although volunteers explained that they also had their issues with other volunteers or visitors, they spoke about the Trading Shop as feeling like “home”.

“What I really appreciated in the Trading Shop is the possibility to get to know new people. We’ve already agreed to go on a walk… that’s nice!”

Next, the domains of spiritual dimension and quality of life were mentioned. They explained that the Trading Shop provided them with something meaningful to do in life, giving them a purpose. This showed that the Trading Shop added something to their own lives, as well as meaning something to another person.

“Due to personal circumstances, I wasn’t feeling well. I was like ‘I must have something to do again’. Then I read the advertisement from the Trading Shop and thought ‘Yes, that’s it…’ Now I mean something to society again.”

With regard to the quality of life, volunteers said they enjoyed their work in the Trading Shop. This helped them to feel good about themselves. Some also stated that the Trading Shop added balance or continuity to their lives.

“The moment I stand behind the counter, I am also the face of the Shop. I like doing that. It helps me to feel more self-assured.”

In addition to these three domains mentioned by almost every volunteer, the domains of mental functions and daily functioning of the positive health tool were mentioned by a smaller group of volunteers. In the domain of mental health, some volunteers explained that the situations they had to deal with in the Trading Shop enabled them to accept themselves more, providing some joy to life. Others explained that they felt somewhat depressed when they were only able to stay at home. Now, they had to go outside, which was helpful.

“What I now notice is that this gives such a boost. Previously I had that with the… group that I supervised. But this is different, now I really have the feeling that I have a job again.”

For daily functioning, practical elements arose, such as learning the Dutch language. The Trading Shop, together with the support of the activation broker, seemed to fulfill the purpose for personal growth among volunteers and provided insights into their talents and qualities.

“I enjoy working with numbers and would like to find a paid job in administration. However, my resume in the Netherlands is empty. The Trading Shop helps me to gain experience and it’s also a place to learn the Dutch language better.”

**Figure 2:**
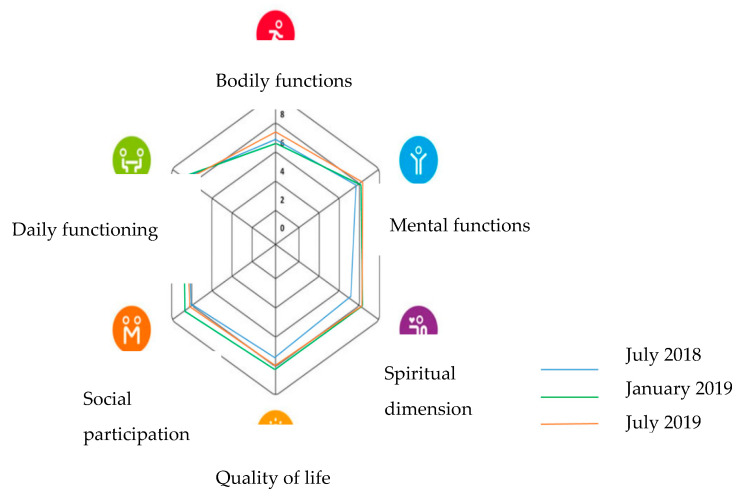
Subjectively rated positive health tool during the interviews with volunteers. Rated from 0 to 10, where each web line in the figure represents an increase of 2 points. In the middle, participants rated the domain with the lowest score 0, and at the edges, the score was 10, indicating that a larger web indicated a better positive health score.

Although some volunteers mentioned that the Trading Shop helped in maintaining their overall vitality, bodily functions were not mentioned in relation to the Trading Shop. Over the study period, we learned that volunteers subjectively rated all six domains of positive health higher compared to the first measurement just after the opening of the Shop (Figure 2).

#### 3.2.2. Engagement by Visitors

About half of the visitors said they became a member of the Trading Shop directly after the festive opening, where others started their membership on a later moment. Fifty percent visited the Trading Shop at least once a week. All visitors mentioned being satisfied with the opening of the Trading Shop. They mostly visited the Trading Shop to trade usable products. The majority explicitly supported the idea of reusing products and appreciated trading without money. Over time, the possibility to meet each other and have free coffee in combination with the nice atmosphere and friendly volunteers seemed to become more important to visitors. The participation of visitors within the community seemed to grow. Their contributions can be divided into: (1) informing each other, including sharing advice and local possibilities to, for example, getting support, (2) undertaking activities together, such as a local hiking group, and (3) providing support, such as offering transportation. We observed that these contributions mostly arose spontaneously. Points for improvements mentioned by visitors were mainly focused on practical things such as more exchanges of products in the shop, fewer intake stops on specific products, a better focus on the quality of products that is accepted, a loading and unloading place for transport nearby the shop to prevent dragging, and more extended opening hours to give employed citizens a chance to visit.

#### 3.2.3. Engaging the Community

Fifteen months after the festive opening, the Trading Shop had 1139 members. About 75% of all members were females. The senior group of above 66 years was the largest, with almost 40%, and the smallest group was aged up to 35 years, with just over 10%. About 15% of all members (170 members) were expected to be a poorer/low-SES family, with children under the age of eighteen. Most members lived in the Netherlands (72.4%), followed by Germany (21.4%) and Belgium (6.2%). Of all members, 60% lived in the Municipality of Vaals. A variety of 28 other Dutch municipalities were represented in the additional 12% of Dutch members. In Germany, Aachen, described as the Big Sister of the Municipality of Vaals, contributed 18.4% of all members.

During the opening hours (four hours per day), the average number of visitors was 62—of whom, about 10 were low-SES families. On a busy day, the Trading Shop had 85 visitors, and the lowest number of daily visitors observed was 45. Every day, about 20 visitors brought products to the shop, and about 25 visitors “bought” one or more items. Together, members traded products and services for a total of 133,126 points in 8757 trading receipts, indicating that over 20,000 products and services were traded. For citizens in a vulnerable position who were not able to trade but would have been supported by the shop, a support fund was set up by the Trading Shop. Members/citizens could also donate products, and the points earned were included in this fund for people in need. They have already donated 15,078 points and used about 2373 points to support citizens in need and societal initiatives. Two to three new visitors became a member of the Trading Shop during every day that was observed. When visitors became members, they earned 15 points for free to immediately start trading. Of all members, 164 (14.4%) still had 15 points on their account as at July 2019, indicating that they probably visited the Trading Shop just once. The other members made use of the Trading Shop to some extent. The mean number of visitors drinking a cup of coffee at the communal table was, on average, 18 visitors per day.

The Trading Shop received support from two structural partners, who provided the groceries needed for the communal table. In addition to these partners, four professionals providing support to citizens in need were available in the Trading Shop at least once a week to support members.

### 3.3. Steps towards Continuation: A Five-Year Policy Plan for Trading Shop Vaals

**Figure 3:**
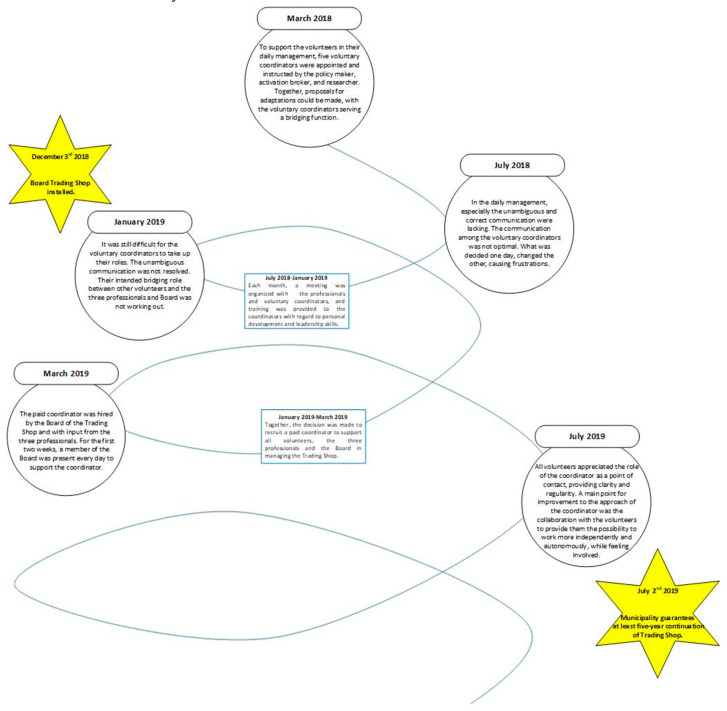
Starting process of the Trading Shop.

After founding the Trading Shop, monitoring cycles were installed to timely support the management and volunteers in running the shop. Many small moments of adjustment took place in the first 15 months. These were mainly practical enhancements that were noticed by volunteers, visitors/members, the researcher, and the broker and discussed together with the daily management. Enhancements focused on reducing the number of products that a member could bring in at one time, rearranging the shop, adding product labels to the shop, obtaining more garden tools, putting additional advertisements on social media, and introducing more rotations in the cleaning scheme. During the first 12 months, these small adjustments were mainly registered by voluntary coordinators and during the interviews/observations of the researcher. All possible adaptations were discussed together with the policy-maker and activation broker before making a decision. An overarching theme in this process was the daily management of the shop, where the lack of unambiguous and correct communication among the voluntary coordinators was described as being problematic. To get the daily management on track, a monitoring cycle was put in place. In addition to all the small adaptations achieved using short feedback loops, several feedback loops had to be passed to enhance this daily management. This adaptation towards efficient and satisfactory daily management was perceived by all partners involved as being the most difficult to get on track to enhance the continuation (Figure 3). In the end, the success of the Trading Shop and the well-organized structure were rewarded by the decision of the municipality to subsidize the Trading Shop for at least the next five years.

## 4. Discussion

The current study aimed to define the value of a Trading Shop as a community engagement approach for low-SES inhabitants of Vaals. The process, output, and outcome of the Trading Shop were monitored from the preparation phase to the steps needed to create a more sustained municipal support, with special attention for community participation. Finally, the value of the Trading Shop was explored in terms of perceived needs and positive health. After the first 15 months, the Trading Shop had engaged 62 volunteers; it had 1139 members and an average of 62 visitors per four-hour workday. This indicates that the shop occupied an important position in the Municipality of Vaals and beyond. The results showed that the Trading Shop focused on the target population’s basic needs of meeting each other and helping each other [19]. Activities within the Trading Shop enhanced the possibility to participate and become meaningfully engaged but, also, to reach out to a broader (vulnerable) community, such as volunteers or members of the Trading Shop.

Although the bottom-up defined idea of preparing and initiating a Trading Shop occurred in the partnership activities with low-SES families, the actual preparation phase was shaped with relatively much top-down input. Some activities had to be carried out within a certain time frame, such as the paperwork but, also, keeping track of the actual organization of the chores that had to be done in the Trading Shop before the festive opening. The deadline was set very strictly by the Municipal Aldermen, indicating only six weeks to get the shop ready. During these “chore weeks”, at least one of the three professionals—the policy-maker, activation broker, or researcher—was always present in the shop to coordinate tasks and do chores. Although the professionals felt they had to push this (vulnerable) group of volunteers too hard to get things done, still, a feeling of ownership and belongingness arose among most volunteers, which became visible after the opening. We learned that, for this preparation phase, an “economic” top-down-oriented approach was successful in achieving quick results [29], making it a suitable and feasible approach for achieving a short-term goal together [30].

Following the festive opening, a focus on “community development” navigating towards a more participatory bottom-up-oriented approach was considered important to achieve community engagement goals [31]. Professionals aimed to let go of their guiding roles, trying to become partners at a little more distance to the Trading Shop. A change in roles was expected to be necessary to enhance an equal partnership. A finer navigation between the top-down approaches and bottom-up approaches became visible, with the aim of making the initiative sustainable. At the start, this was perceived to be difficult, since appointed voluntary coordinators were not trained to manage a shop, making their communication unambiguous to other volunteers. Additionally, the work formats used by the professionals to collaborate with the voluntary coordinators, e.g., mails and meetings, did not work properly [20]. After several months of attempts to train and organize the daily management, everyone agreed upon installing one paid coordinator for the whole shop. This process showed that some top-down influence needs to be present in a shop, since a trained and paid coordinator present in the shop could get “things” done to achieve societal goals and work towards sustainability [32]. We learned that it is important for professionals in these community engagement approaches to work towards meaningful engagement. In these approaches, different partners and their powers and interests have to be dealt with while maintaining key elements for community engagement to be successful, such as trust, equity, accessibility, contextualization, and transparency to enhance partnership [12,13,14,15,16,17,18,33].

Various societal objectives came together in participatory activities of the Trading Shop. These included facilitating opportunities to meet and help yourself and others, increasing and retaining independence and resilience, combating waste, and alleviating financial problems. Constant feedback loops were in place to facilitate this process, enhancing the navigation between the top-down and bottom-up inputs. In particular, social participation and activation was mentioned by volunteers as being the most important contribution of the Trading Shop to their own lives and to the lives of visitors. During interviews, the enhancements in their social network and having a sense of belongingness were said to be the most important personal additions of the Trading Shop. Volunteers called the Trading Shop a “home”, showing the importance of a strong social network [34]. Additionally, the spiritual dimension and the quality of life were explicitly mentioned, showing the importance of being meaningfully engaged. Interestingly, other domains also increased, while volunteers were less aware of them [21]. The inclusivity and accessibility of the Trading Shop as a community engagement initiative offered the opportunity to volunteers to fulfill their needs concerning personal growth and the development of their talents in several positive health domains.

### 4.1. Strengths and Limitations

A major strength of this study was the participatory design that provided the opportunity for the researcher to develop a close collaboration with everyone involved in the Trading Shop, such as the volunteers, members, and partners. This was found helpful in gaining insight into the engagement of the community at a specific point in time, making timely adaptations possible. Second, the inclusion of the process, output, and outcome data using various quantitative and qualitative sources were helpful in creating an overview of the added value of the Trading Shop to the community. Although we had some privacy constrictions regarding gathering data about the reach of our target group as members of the Trading Shop, in conjunction with the observations, we were able to estimate the number of families supported by the Trading Shop.

This study also had some limitations. First, due to the dynamic context and the open research design, it was hard to define the actual contributions of the Trading Shop to the volunteers and members. We used the positive health tool [21] to obtain more insight into the personal development of the volunteers. The positive health score is not a validated tool, and we were not able to test the progress of the volunteers due to the small sample. We had to use absolute shifts in the positive health web as an indication of the volunteer progress. However, we still found it helpful to provide an idea of the processes that occurred in the Trading Shop, especially based on the qualitative research features of the tool. Secondly, the generalizability may be limited, since the Trading Shop was established in only one municipality. We aimed to increase the generalizability by adopting a helicopter view to reflect on guiding principles underlying the processes in the Trading Shop and to extract learnings for future participatory approaches.

### 4.2. Future Research and Practice

Many community engagement initiatives are undertaken with the aim of creating equal partnerships. More insight in these actual partnerships within the broader community are required to better understand the processes that underly successes and failures in community engagement initiatives [35]. We encourage researchers to be flexible with respect to local opportunities and to carefully monitor both processes and effects. The sustainability of community engagement initiatives should be granted a major point of focus in the collaboration between research, practice, and policy.

In practice, we encourage professionals working in community engagement activities to take steps towards collaboration and empowerment [4]. In order to achieve community engagement, it is important to start taking action and work together on topics useful to the community to eventually enhance their well-being [36]. Striving for results and making ideas tangible [29] was found to be beneficial to engage the community. Finally, continuously navigating top-down and bottom-up approaches is needed to achieve results.

## 5. Conclusions

The Trading Shop seemed to be a promising community engagement initiative in satisfying various societal needs of low-SES families. For the vulnerable community in the present study, the Trading Shop was found to be particularly successful in facilitating opportunities for social participation and activation. A key promoting factor for the community engagement activities of the Trading Shop is the optimal navigation between top-down steering from professional coordinators and bottom-up-oriented activities from citizens. Overall, the Trading Shop can be seen as a sustainable connecting factor within the Municipality of Vaals.

## Figures and Tables

**Table 1:** Observation list. SES = socioeconomic status.

Number of visitors
Number of visitors drinking a cup of coffee at the communal table
Number of visitors bringing products to the Trading Shop
Number of visitors “buying” products from the Trading Shop
Number of visitors in the target group (low-SES) families
Notes about remarks of visitors/members and volunteers

**Table 2:** Interview guide visitors/members.

Question 1: Why do you visit the Trading Shop?
Question 2: Since when have you been visiting the Trading Shop?
Question 3: How often do you normally visit the Trading Shop?
Question 4: What do you like about the Trading Shop?
Question 5: Do you have any ideas to improve the Trading Shop?

**Table 3:** Interview guide volunteers.

Question 1: When did you start as a volunteer at the Trading Shop?
Question 2: How many days a week do you volunteer at the Trading Shop?
Question 3: What is your task within the Trading Shop?
Question 4: What do you like most about the Trading Shop?
Question 5: Did the Trading Shop support you personally? How?
Question 6: Do you have any ideas to improve the Trading Shop?

**Table 4:** Interview guide group interviews.

How have you experienced volunteer work at the Trading Shop?
What do you like, and what could be improved?
Looking at the future, what would you like to focus on in the next six months to enhance the Trading Shop?
